# Do locking plugs improve implant strength? Biomechanical comparison of polyaxial locking constructs with and without locking plugs in a fracture gap model

**DOI:** 10.1186/s12917-023-03660-x

**Published:** 2023-08-02

**Authors:** Joni Viitanen, Robert Quinn, Matthew Allen, Bart J. G. Broeckx, Tomasz Bartkowiak, Georg Haimel

**Affiliations:** 1 Veterinary Specialists Scotland, part of Linnaeus veterinary limited, Livingston, EH54 8AG United Kingdom; 2 Anderson Moores Veterinary Specialists, part of Linnaeus veterinary limited, Hampshire, S021 2LL United Kingdom; 3 Department of Veterinary Medicine, Surgical Discovery Centrum, University of Cambridge, Cambridge, CB3 0ES United Kingdom; 4 Department of Nutrition, Genetics and Ethology, Faculty of Veterinary Medicine, Ghent University, 9820 Merelbeke, Belgium; 5 Institute of Mechanical Technology, Poznan University of Technology, 60-965 Poznań, Poland; 6 Tierarztpraxis Am Stadtpark, Reisnerstrasse7, 1030 Vienna, Austria

**Keywords:** Polyaxial locking plate, Locking plug, Implant failure, Bending, Fracture model

## Abstract

**Background:**

The objective of this study was to investigate the effects of locking plugs and the biomechanical properties of a 3.5 mm 8-hole polyaxial locking plate in a fracture gap model. Our hypothesis was that locking plugs would increase the strength and stiffness of the construct. Twelve 3.5 mm 8-hole plates were used to evaluate two different construct designs (with locking plugs vs. without locking plugs) with validated bone substitutes in a 25 mm bridging osteosynthesis gap model. Each construct was subjected to a single cycle four-point bending load to failure using a servo-hydraulic testing machine. Bending stiffness, bending strength, and bending structural stiffness were calculated and compared using an unpaired Student´s t-test.

**Results:**

The plating construct with locking plugs did not show any significant increase in terms of bending stiffness, bending strength, and bending structural stiffness compared to plating construct without locking plugs in a 25 mm gap fracture model during a single cycle four-point bending.

**Conclusions:**

Under the conditions tested, filling empty plate holes with locking plugs in bridging osteosynthesis does not increase stiffness or strength of the plate-bone construct.

## Introduction

Biologic plate osteosynthesis has become an increasingly popular technique for internal fixation of fractures in small animals [1, 2]. Bridging comminuted fractures with a long bone plate without disrupting the fracture hematoma has been shown to be advantageous with regards to bone healing and early return to function [3]. Locking plates are used for treating comminuted fractures using a MIPO (minimal invasive plate osteosynthesis) or “open but do not touch” technique for fracture stabilization. Locking plugs are used in various locking plate systems to avoid distortion of the screw holes during plate contouring. Currently there are no recommendations whether locking plugs should be removed or left in place after plate bending or even added intentionally to fill empty holes during bridging osteosynthesis to improve stiffness and fatigue life of the construct. Contradictory results exist as to whether or not locking plugs should be removed after plate contouring or should be left in situ to decrease stress concentration and increase construct strength and stiffness [4–12]. Several previously published studies failed to demonstrate a significant difference in stiffness, torsional strength, or axial loading strength of the locking plates with the addition of screw hole plugs [4, 5, 9, 12]. However, in the study by Bellapianta et al. filling the open hole with a screw head reduced the stress at the periphery of the hole and increased the stiffness of the plate. Further, a fourfold improvement in fatigue life when a screw plug was added to the empty holes of the plate was found in the same study [6]. The magnitude of these effects varied between plates from different manufacturers. The authors of the same study further hypothesized that the design, material and the magnitude of the loads to which the plate is subjected can influence the effect of locking plugs filling empty screw holes. The purpose of our study was to compare a stainless steel polyaxial locking plate (PLP) (EVOLOX®, N2 (UK) Ltd, Portsmouth, UK) constructs with and without locking plugs during a single cycle load in four-point bending until failure. We hypothesized that, as the plates consist of circular screw holes, a construct with locking plugs that completely fill these would have a higher stiffness and strength. The locking plugs would be expected to stiffen the plates when applied as they should theoretically lock into the plate and act as a single construct, thereby increasing the area moment of inertia at the screw holes and the stiffness of the plates in a bridging osteosynthesis model.

## Material and methods

This study was performed in accordance with appropriate guidelines with no animals involved.

### Area moment of inertia

The AMI of the plate and plug was calculated using a commercially available software^1^ based on the 3D models created from the measurements of real physical geometry of those objects. Area moment of inertia is a mathematical representation of the distribution of material about an axis through the centre of an object's cross-section [13]. These values were calculated at the location of the plate holes (empty) and between holes (solid section). Two values for the plug: maximum and minimum, were calculated due to the complex geometry of the star socket and the fact that angular orientation of a plug with a reference to a plate hole is usually random.

### Construct assembly

**Fig. 1:**
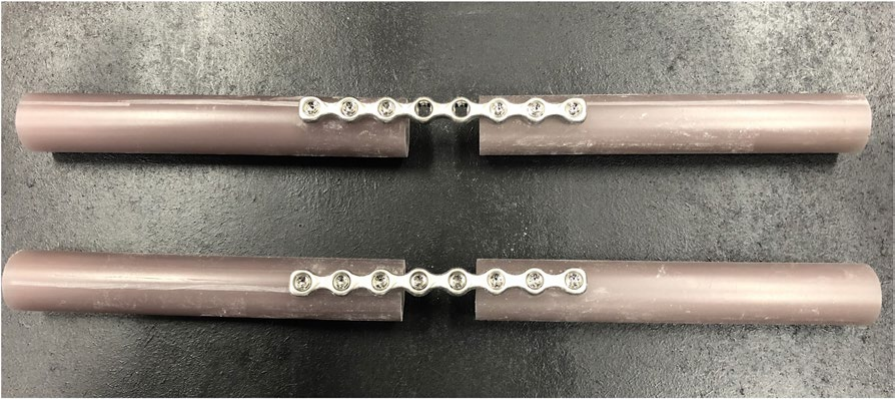
Plate constructs without (top) and with (bottom) locking plugs

A fourth-generation bone model consisting of short-fibre filled epoxy hollow cylinders, with a uniform cross-sectional geometry of 20 mm in diameter and 3 mm wall thickness was used to simulate the bone segments^2^ [14, 15]. The cylinders were cut into pieces 125 mm in length. The segments were aligned using a specially designed custom jig leaving a gap of 25 mm between the two cylinders. A bone plate was centred on the gap, aligned over the bone segments and held in place by locking bridges on the jig. Twelve eight-hole, 3.5-mm stainless steel polyaxial locking plates^3^ with 3.5-mm self-tapping locking screws^3^ were used for this study. The constructs were divided into two groups: each with 6 specimens (without locking plugs) and (with locking plugs) (*n* = 6/plate type). In both groups, screw holes were drilled with a 2.8 mm drill using a locking drill guide and each plate was secured using six (3 per bone model segment) 3.5 mm bicortical locking screws, inserted perpendicular to the plate. To provide homogeneity and repeatability, the insertion torque applied to each screw was standardised to 1.5 Nm using a torque-limiting screwdriver^4^, as recommended by the manufacturer. The spacer was removed, resulting in final plate-constructs with a 25 mm gap, simulating a bridging osteosynthesis (Fig. 1).

### Mechanical testing

**Fig. 2:**
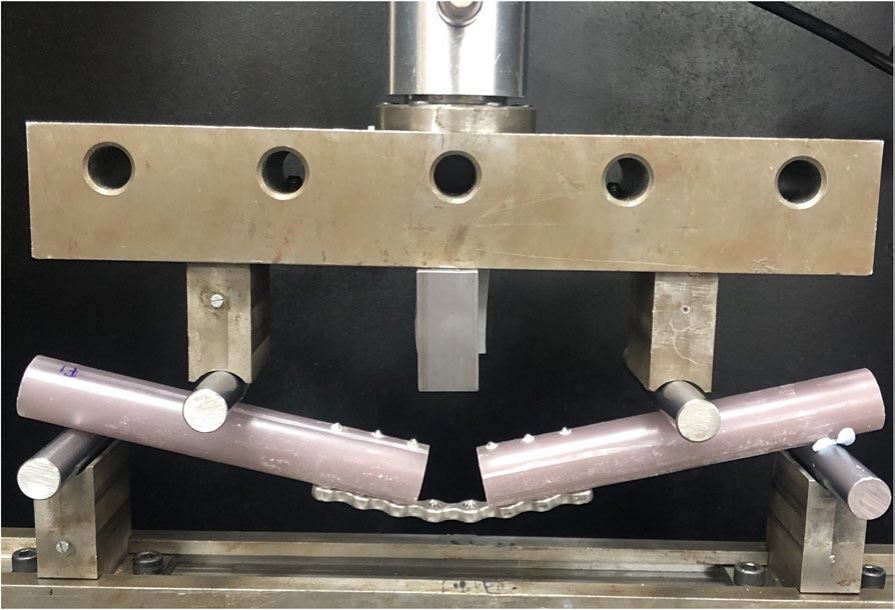
Mechanical testing configuration for four-point bending used for plate-constructs as situated in the servo- hydraulic testing machine

**Fig. 3:**
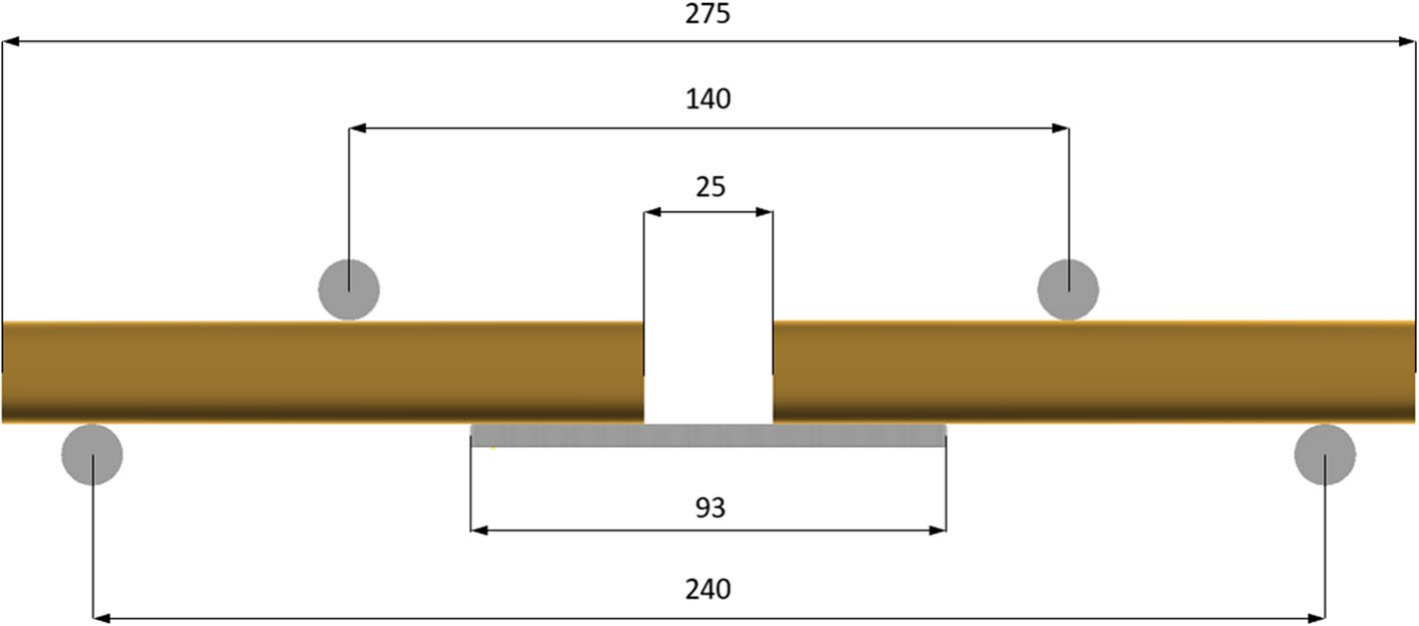
Mechanical testing configuration for four-point bending used for plate-constructs as situated in the servo- hydraulic testing machine

Destructive static tests were performed using a universal servo hydraulic testing machine^5^. Our mechanical testing protocol was based on previous studies (Blake & Eid). All plate constructs were manually centred on the support rollers with the portion of the plate with the minimum section modulus facing the loading direction and subjected to a four-point bending test with a monotonic single ramped load to failure in accordance to the ASTM Standard Specification and Test Method for Metallic Bone Plates [16]. Tests were carried out under displacement control, with loading rate of 0,1 mm/s using a load cell of 2kN. Displacement between the top and bottom parts of the 4-point bend loading rig was measured using a laser extensometer^6^ with a repeatability of 0.005 mm. Data was collected using at a frequency of 10 Hz. The loading roller diameters were 12 mm. Centre span = *a* and loading span = *h* remained constant for all samples in both groups with the centre span and loading distances being 140 mm and 50 mm. The 0.2% offset displacement of 0.28 mm was used, as 0.002 × a, where a is the centre span distance. Displacement control was used with a loading rate of 0.1 mm/s. All measurements were made using a Vernier calliper^7^(Figs. 2 and 3).

Bending stiffness was calculated by determining the maximum slope of the elastic portion of the load versus load-point displacement curve (N/mm), which was determined using linear regression analysis for a best fit. The gradient (K) was used to determine the bending structural stiffness with the equation:

$$\mathrm{Bending}\;\mathrm{structural}\;\mathrm{stiffness}=\mathrm{EI}=((2\mathrm h+3\mathrm a)\;\mathrm{Kh}2)/12$$

where E = Young’s modulus, I = area moment of inertia, a = centre span distance (distance between inner supports), and h = loading span distance (distance between the inner and outer support).

Bending strength (yield point) is defined as the bending moment needed to produce a 0.2% offset displacement (plastic deformation) in the bone plate [16]. The corresponding yield (proof) load was converted to a bending strength (moment) using the load span distance (h) for each specific test using the equation:

$$\mathrm{Bending}\;\mathrm{strength}=(\mathrm{Ph})/2$$

where P = proof load and h = loading span distance. Bending structural stiffness is defined as the bone plate’s normalized effective bending stiffness that takes into consideration the effects of the test setup configuration [16]. Therefore, as bending structural stiffness of the bone construct is related to the geometry of the bone plate and the material used in manufacturing the bone plate, it is an indicator of the stiffness of the bone plate itself, independent of the test configuration. Bending strength depends on the area moment of inertia.

Bending moment deflection curves were generated using commercially available software^8^. Bending stiffness, bending structural stiffness and bending strength were calculated for all constructs.

### Statistical analysis

A priori, a power analysis was performed in R to determine the minimum sample size with the following parameters: unpaired two-sample student’s t-test, α = 0.05/3 (to correct for multiple testing) and power = 0.8. Furthermore, the effect size was calculated based on the idea that a difference of 20% in bending stiffness would be clinically relevant (i.e., a difference of 0.2µ_1_ with µ_1_ based on earlier studies [14, 17]. Taking earlier published average values and their standard deviations into account, the effect size was set to 2.4. Based on these settings, a sample size of 6 per group was found to be sufficient.

The data from the two groups (filled and unfilled) was compared with the unpaired two-sample student’s t test. Significance was set at α ≤ 0.05. A Bonferroni-correction was applied to correct for multiple testing and only corrected *p*-values are reported.

## Results

### Area moment of inertia

**Fig. 4:**
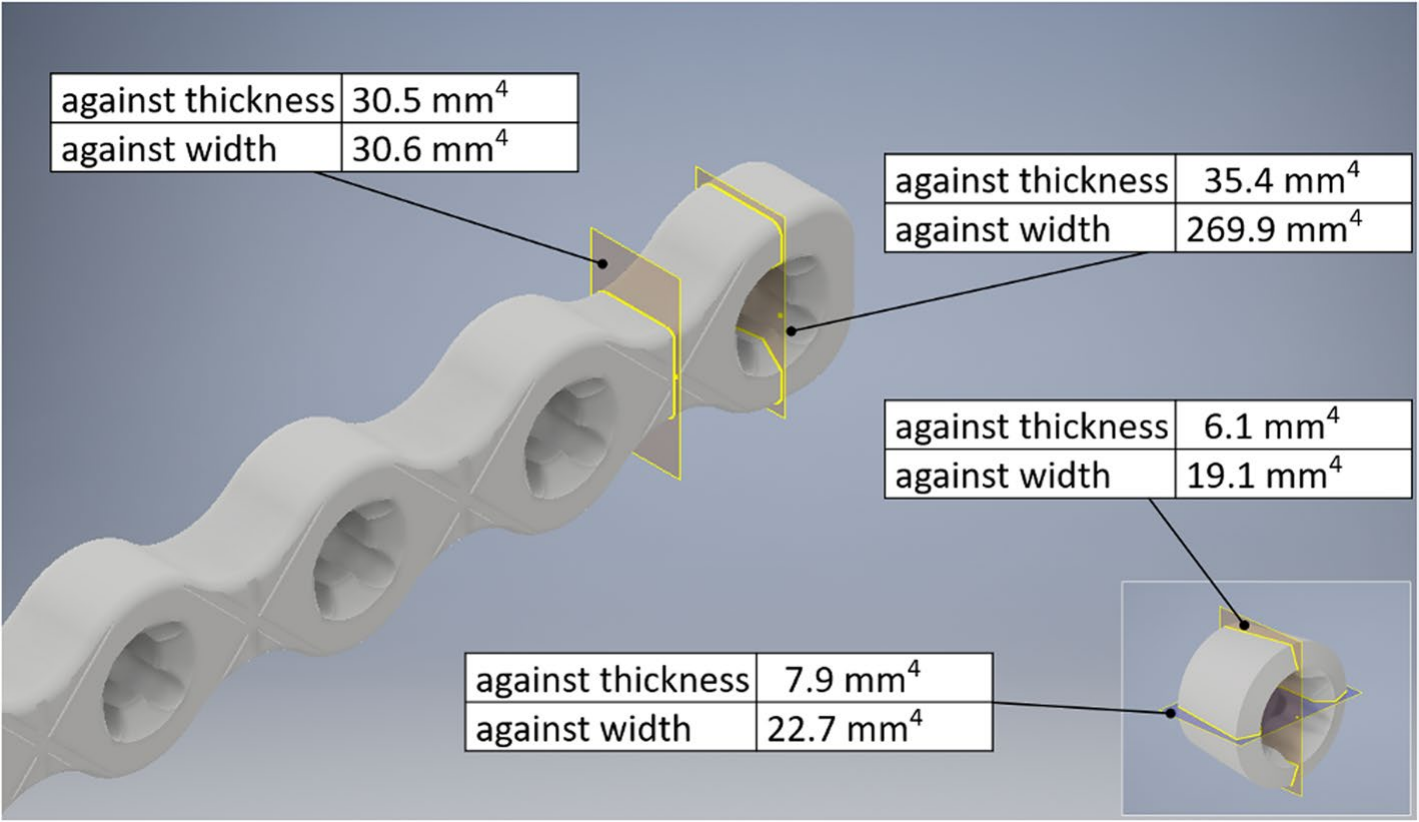
Schematic illustrations of cross sectional profiles of the polyaxial locking plate and locking plug used in this study, both at the level of the plate holes, filled plate holes and between holes. Area moment of inertia are calculated based on the yellow portions of the cross-sections

The AMI when loaded against its thickness is 35.4 mm^4^ (plate hole) and 30.5 mm^4^ (solid section). All other relevant AMI values are presented in Fig. 4.

### Four point bending single cycle to failure

**Fig. 5:**
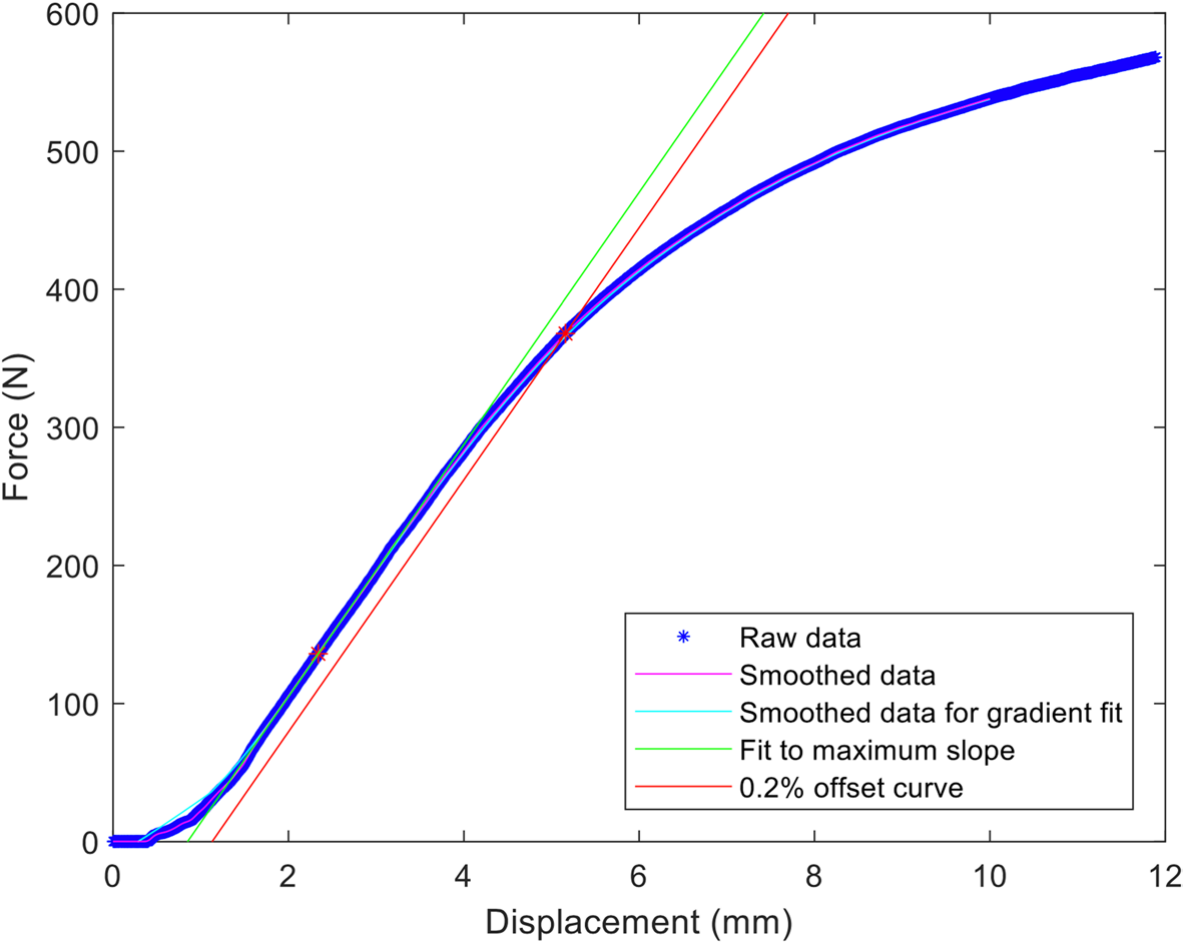
Analysis of force–displacement curve

All of the construct models failed by plastic deformation of the plate within the region of the gap between the bone model cylinders; no implant breakage occurred. Data was analysed using a bespoke programme written using Matlab software following the ASTM methodology. The bending stiffness was calculated from the maximum slope of the load–displacement curve, using a smooth fit to eliminate the effect of noise or scatter in the data. Bending stiffness, bending strength and bending structural stiffness are calculated following the ASTM standard (Fig. 5). During the test, all of the plates reached a plastic deformation of 0.2%. Results of one construct with plugs was excluded as there was a significant jump in the displacement close to the maximum slope and a corresponding drop in load, presumably associated with some settling effect of the screws. There were no significant differences between the two constructs for bending stiffness (*p*-value = 1), bending strength (*p*-value = 1) or bending structural stiffness (*p*-value = 1) in a single cycle load to failure (Table 1).

**Table 1:** Summary of plate construct data (mean ± standard deviation)

Construct	Bending stiffness (N/mm)	Bending strength (Nm)	Bending structural stiffness (Nm^2^)
Unfilled	92.22 ± 6.6	8.58 ± 0.2	9.99 ± 0.7
Filled	92.0 ± 6.5	8.74 ± 0.8	9.96 ± 0.7

## Discussion

**Fig. 6:**
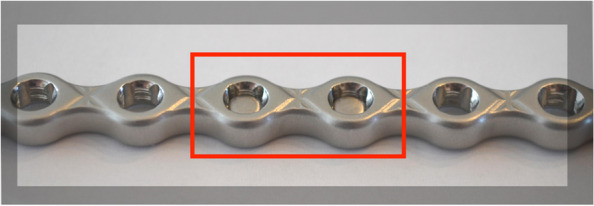
Locking plug inserted in the screw hole. Note the holes not being fully filled by the locking plugs

We investigated the effects of locking plugs on the bending properties of a stainless steel polyaxial locking plate system in a single cycle four-point bending load to failure model. AMI is an important part of biomechanics, as this parameter can be directly compared with other plates like Locking Compression Plate (LCP), Dynamic Compression Plate (DCP), String of Pearls (SOP) etc. The differences between AMI, what results in theoretical bending stiffness and strength as well as the orientation of the plate on the bone, should be considered when planning an internal fixation construct. The AMI, for our tested plate, when loaded against its thickness is 35.4 mm^4^ (plate hole) and by 16% larger when compared to the solid section. However, while filling the empty holes increases the “metal mass”, we did not observe an increase in stiffness and strength properties. A possible reason for our test results is that the locking plugs measured 3 mm in thickness, whereas the plate thickness was 4.5 mm. Therefore, a small portion of each plate hole was not filled by the locking plug, meaning the locking plugs do not balance or increase the thickness of the plate, which is an important factor contributing to the stiffness and strength of the implant (Fig. 6).

Further, the portion of the plug, that transmits load and thus contribute to the mechanical properties of the construct is limited to only the threaded part which is in physical engagement with plate thread.

AMI strongly depends on the distance between portion of the material that transmits load and the gravity centre of the cross-section area. The further the distance to the gravity centre the higher the AMI. When bending against thickness, the threaded part of the plug is close to the gravity centre what causes that the beneficial effect of the locked plug may be marginal to improving bending strength and stiffness. Bellapianta and colleagues used a fracture 5 mm gap model with a solid synthetic bone in four point bending and found an increase of stiffness with 26.4% and fourfold improvement in fatigue life with adding a screw head in empty locking hole plates during a cyclic four point bending [6]. As mentioned above a possible explanation for this observation could be that a) the thickness of the screw head was closer to the plate thickness and b) that screw heads have a larger area which actively transmits forces compared to locking plugs. Consequently, a different design of locking plugs might be necessary to improve the biomechanical properties of locking plates when large areas of comminuted bone defects must be bridged.

With regards to the screw/plate interface, the screw head and the locking plugs both use a thread to thread coupling as locking mechanism. We standardised the screw and locking plug insertion torque at 1.5 Nm, as recommended by the manufacturer, which was at the lower end compared to previous studies [9, 11, 18]. Thread engagement grows with the insertion torque what results in an increased volume of metal, that actually transmits the load. It has been shown that increasing the insertion torque of locking screws can improve the fatigue performance [8, 10, 19]. Meyers and colleagues showed increased bending stiffness in bone plates when tightening the inserts to twice the recommended insertion torque in 3-point bending under cyclic loading. However, there is no recommendation how tight locking plugs should be inserted into the screw holes. In a clinical setting they are usually not tightened as removal after plate bending can be challenging. If locking plugs are planned to remain in the plate an increased insertion torque could potentially positively influence the biomechanical behaviour of the construct.

As previously published, plate-bone constructs may exhibit a different mechanical behaviour than a plate alone, even more so if high bending moments are used, and if constructs are tested to failure. Blake and colleagues showed plate-bone constructs having lower stiffness than the plates alone [14]. Plate-bone construct testing is therefore an essential step in the mechanical evaluation of an implant. Our study used a fourth-generation synthetic bone models for creation of the testing constructs to minimise inconsistency in shape that would be present in cadaver bone. The synthetic bones are validated for use in biomechanical testing; however, they do not perfectly mimic the biomechanical properties of bone and may have greater resistance to screw pull-out or fracture but this is unlikely to influence the results of this study. Alternative models include cadaveric bone and solid synthetic acetal rods as bone substitutes [6, 11, 20]. A discrepancy in results has been also suggested in different studies, where different bone material was used [17, 21]. However, the variation in structure and dimension of a cadaveric bone, which can also be challenging to obtain, makes it impractical for testing individual constructs. Nevertheless, we cannot completely exclude that the constructs would have behaved differently if a different bone substitute would have been used.

A 25 mm fracture gap was selected for our testing as this has been used in previous studies evaluating biomechanical properties of bridging plates and is also a common clinical scenario for the tested plate size [14, 17]. Given the direction with which the load was applied during the mechanical testing, the working length was equal to the length of the fracture gap [20, 22]. The gap model simulates comminution by preventing cortical contact between the two fragments. In prior non-gap models, axial deformation permits cortical contact between the two fragments. This would potentially alter our results, as we were seeking to evaluate the biomechanical properties of just the implants.

Our study tested single-plate constructs as they are commonly used clinically and are most at risk of failure [9]. The protocol was based on the methodology of previously reported studies, where testing was performed in four-point bending, under similar testing conditions [14, 17, 11, 23]. Our plate was in direct contact with the bone substitute. Increasing the bone-plate stand-off distance greater than 2 mm might have influenced the bending moment of the construct and reduced the mechanical performance as shown in other studies [11, 22].

In our study no significant differences between the two constructs for bending stiffness, bending strength, or bending structural stiffness were found. Our hypothesis, that the locking plugs lock into the plate and act as a single construct and thereby increasing the area moment of inertia at the screw holes and the stiffness of the plates was thus not confirmed.

Previous studies have yielded contradictory results when locking plugs are added to a locking plate. Hung’s et al. study demonstrated no difference in stiffness when testing a bone plate under single four point bending but found a 106% increase in fatigue life during cyclic fatigue tests for stainless steel plates but no difference for titanium plates [7]. Carter et al. used a four-point bending testing setup of a stainless steel implant solely [8]. Their results showed increased fatigue life of locking plates, especially when increasing the screw torque of the locking plugs. Our study solely examined the biomechanical properties in a single cycle four point bending rather than a cyclic four point bending model which is a limitation of our study and do not represent all forces in the clinical scenario.

Additional mechanical testing such as cyclic fatigue failure tests, torsion tests, and biplanar bending tests of the same constructs may be of interest and could potentially demonstrate a benefit of the locking plugs as published in the above mentioned studies.

Acute mechanical failure is a concern in bridging osteosynthesis where all forces are transmitted by the implant. According to Bertram et al., the force applied to hindlimbs for a 40 kg dog while running at a trot would be ~ 297 to 419 N (76–107% of body weight) [24]. Under single cycle bending loading, plastic deformation for the PLP occurred at 343.2 N without locking plugs and at 349.6 N with locking plugs. This highlights the importance of cage rest and restricted activity. Additionally, Kaczmarek and colleagues reported a polyaxial locking system 30% weaker in bending stiffness, bending strength and bending structural stiffness than a locking compression plate [23]. This may suggest that using a PLP plate alone should be done with caution in clinical situations where the construct is subjected to higher loads. This common clinical scenario would benefit from the ability to increase the plate strength whilst still having the advantages of being polyaxial locking plate system, for example by adding an intramedullary pin or a second plate.

## Conclusion

Our results indicate that the added locking plugs used in our model of a single 4-point bending loading do not offer clinically relevant benefits in terms of strength and yield load compared to the locking plating construct without locking plugs, in a 25 mm gap fracture model. It is very important to consider that pure four-point bending loading rarely occurs in vivo and therefore the results of this experimental study are not totally relevant to clinical situations. Additional studies including cyclical testing and testing different plug designs would be desirable to further enhance our knowledge about the potential clinical benefit of locking plugs in clinical application.

## Data Availability

https://osf.io/n7q86/.

## Abbreviations

AMI: Area Moment of Inertia
DCP: Dynamic Compression Plate
LCP: Locking Compression Plate
MIPO: Minimally Invasive Plate Osteosynthesis
PLP: Polyaxial Locking Plate
SOP: String of Pearls SOP

## Footnotes

1 Autodesk Inventor; Autodesk Inc., USA

2 Sawbones, Pacific Research Laboratories, Inc., Vashon Island, WA, USA

3 EVOLOX ®, N2 (UK) Ltd, Portsmouth, UK

4 Felo Werkzeugfabrik Holland-Letz GmbH

5 Instron 6025 processor, High Wycombe, UK

6 EIR, model LE-05, Irwin, PA, USA

7 Clarke Ltd. UK

8 Microsoft Excel, Microsoft Corporation, Redmond, USA
